# Structural comparison of allophycocyanin variants reveals the molecular basis for their spectral differences

**DOI:** 10.1007/s11120-023-01048-4

**Published:** 2023-09-29

**Authors:** Christopher J. Gisriel, Eduard Elias, Gaozhong Shen, Nathan T. Soulier, Gary W. Brudvig, Roberta Croce, Donald A. Bryant

**Affiliations:** 1 Department of Chemistry, Yale University, New Haven, CT 06520 USA; 2 Department of Physics and Astronomy and Institute for Lasers, Life and Biophotonics, Faculty of Sciences, VU University Amsterdam, 1081 HV Amsterdam, Netherlands; 3 Department of Biochemistry and Molecular Biology, The Pennsylvania State University, University Park, PA 16802 USA; 4 Department of Molecular Biophysics and Biochemistry, Yale University, New Haven, CT 06520 USA; 5 Present Address: Department of Biology, University of California San Diego, La Jolla, CA 92093 USA

**Keywords:** Phycobiliprotein, Photosynthesis, Low-light photoacclimation, Far-red light photoacclimation, Phycocyanobilin, Energy transfer, Exciton coupling

## Abstract

Allophycocyanins are phycobiliproteins that absorb red light and transfer the energy to the reaction centers of oxygenic photosynthesis in cyanobacteria and red algae. Recently, it was shown that some allophycocyanins absorb far-red light and that one subset of these allophycocyanins, comprising subunits from the ApcD4 and ApcB3 subfamilies (FRL-AP), form helical nanotubes. The lowest energy absorbance maximum of the oligomeric ApcD4-ApcB3 complexes occurs at 709 nm, which is unlike allophycocyanin (AP; ApcA-ApcB) and allophycocyanin B (AP-B; ApcD-ApcB) trimers that absorb maximally at ~ 650 nm and ~ 670 nm, respectively. The molecular bases of the different spectra of AP variants are presently unclear. To address this, we structurally compared FRL-AP with AP and AP-B, performed spectroscopic analyses on FRL-AP, and leveraged computational approaches. We show that among AP variants, the α-subunit constrains pyrrole ring A of its phycocyanobilin chromophore to different extents, and the coplanarity of ring A with rings B and C sets a baseline for the absorbance maximum of the chromophore. Upon oligomerization, the α-chromophores of all AP variants exhibit a red shift of the absorbance maximum of ~ 25 to 30 nm and band narrowing. We exclude excitonic coupling in FRL-AP as the basis for this red shift and extend the results to discuss AP and AP-B. Instead, we attribute these spectral changes to a conformational alteration of pyrrole ring D, which becomes more coplanar with rings B and C upon oligomerization. This study expands the molecular understanding of light-harvesting attributes of phycobiliproteins and will aid in designing phycobiliproteins for biotechnological applications.

**Supplementary Information:**

The online version contains supplementary material available at 10.1007/s11120-023-01048-4.

## Introduction

Cyanobacteria and red algae produce phycobiliproteins (PBPs) to extend the number and wavelength range of photons used for the light reactions of oxygenic photosynthesis (Glazer 1989; Sidler 1994; Bryant and Canniffe 2018). PBPs vary widely among organisms and within organisms their expression is often governed by environmental conditions (Bryant 1982; Ho et al. 2017b). There are three major types of PBPs: red-colored phycoerythrins, blue-colored phycocyanins (PC), and aqua-colored allophycocyanins (AP; Greek, “other algal blue pigment”) (Bryant and Canniffe 2018). PBPs bind linear tetrapyrrole chromophores (bilins) for light harvesting that typically absorb in the 500–650 nm range (Ledermann et al. 2017; Bryant et al. 2020a), and they self-assemble into diverse complexes termed phycobilisomes (PBS) (Bryant et al. 1979; Chang et al. 2015; Zhang et al. 2017; Ma et al. 2020; Zheng et al. 2021; Domínguez-Martín et al. 2022; You et al. 2023). PBS form interactions with both photosystem I and photosystem II so that energy absorbed by the PBPs can be transferred to both photosystems to drive photochemistry optimally (Bryant and Canniffe 2018).

AP typically occur as several variants in any given cyanobacterium, and these variants have important, specialized functions in light harvesting and excitation energy transfer as well as structural functions in the assembly of the core substructures of PBS. The major AP variant, which will herein simply be referred to as AP, is the product of the *apcA* and *apcB* genes, comprises a heterodimeric protomer (αβ) that oligomerizes to form toroid-shaped trimers (αβ)_3_, and absorbs maximally at ~ 650 nm. Note that heterodimeric protomers of PBP are usually referred to as “monomers” in the literature. AP-B is an important but minor AP variant that assembles with the same β-subunit (ApcB) found in AP; however, the α-subunit is the product of the *apcD* gene. AP-B has a broad absorbance band at ~ 618 nm and a narrow, more intense absorbance band at ~ 670 nm (Glazer and Bryant 1975; Lundell and Glazer 1981; Peng et al. 2014). AP-B is one of two so-called terminal emitters that occur in the PBS core (Bryant and Canniffe 2018), and it is specifically associated with energy transfer to photosystem I (Zhao et al. 1992; Gindt et al. 1994; Dong et al. 2009; Liu et al. 2013). ApcF is a minor variant AP β-subunit, sometimes called β^18^, which absorbs maximally at ~ 618 nm (Bryant et al. 1990). ApcF forms specific structural interactions near the chromophore of the bilin-binding domain of the other terminal emitter in PBS, the core-membrane linker protein known as ApcE, which is involved in energy transfer to photosystem II (Glazer 1989; Zhao et al. 1992; Dong et al. 2009; Chang et al. 2015; Zheng et al. 2021). Although uncommon, some cyanobacteria produce other variant AP subunits (e.g., ApcA2 and ApcB2), whose roles in PBS structure and function are still poorly understood. Finally, in recent years, it has become clear that some cyanobacteria produce AP variants that absorb far-red light (FRL-AP). These FRL-AP have distinctive α-subunits that appear to have evolved by gene duplication and divergence from ApcD, and they are denoted ApcD2, ApcD3, ApcD4, and ApcD5 (Gan and Bryant 2015). These FRL-AP variants partner with variant β-subunits, either ApcB2 or ApcB3. FRL-AP play important roles in two photoacclimation responses: low-light photoacclimation (LoLiP) (Soulier et al. 2022) and far-red light photoacclimation (FaRLiP) (Gan et al. 2014; Li et al. 2016; Ho et al. 2017a; Bryant et al. 2020b).

PCs and all AP variants bind the same linear tetrapyrrole (bilin) chromophore, phycocyanobilin (PCB), but the absorbance maxima of these proteins and their subunits occur over a wavelength range spanning ~ 115 nm (600 to 715 nm) (Sidler 1994; Bryant and Canniffe 2018; Soulier et al. 2020). Therefore, the absorbance properties of PCB must be extensively tuned by the protein environments in which this bilin is bound (Glazer 1989; Sidler 1994; Bryant and Canniffe 2018; Soulier et al. 2020). In hemidiscoidal PBS, AP and the minor AP variants AP-B and ApcF form torroid-shaped trimers, which stack to form cylindrical cores bound primarily to photosystem II. The organization of the core is scaffolded and directed by the core-membrane linker PBP, ApcE (Bryant and Canniffe 2018). Protomeric (αβ) AP absorbs maximally at ~ 615 nm and protomeric (αβ) AP-B at ~ 621 nm. Upon oligomerization to form (αβ)_3_ trimers, the absorbance spectra of these two AP variants each split into two bands, one of which retains an absorbance maximum at ~ 615 nm but the other red shifts ~ 25–30 nm. The molecular basis for this red shift is unclear, and several possibilities have been discussed in the literature. Recently, it was discovered that low-light ecotypes of some thermophilic *Thermostichus* species (formerly *Synechococcus*; (Strunecký et al. 2023)) produce FRL-APs (ApcD4-ApcB3) that assemble as helical nanotubes instead of torroidal complexes and that they probably bind to photosystem I (Nowack et al. 2015; Olsen et al. 2015; Soulier et al. 2022; Gisriel et al. 2023). Whereas trimeric AP and AP-B exhibit red-most absorbance maxima at ~ 650 and 670 nm, respectively, the absorbance maximum of FRL-AP occurs at 709 nm. The availability of structural and spectroscopic data on multiple AP variants provides an opportunity to explore the molecular bases for the differences in their spectral features and to investigate the origin of the 30-nm red shift that occurs ubiquitously upon oligomerization of the protomers of AP variants.

In this study, we compared the structures of AP (ApcA-ApcB), AP-B (ApcD-ApcB), and FRL-AP (ApcD4-ApcB3) to provide insights into the differences in their spectral features. We describe unique steric interactions found in the α-subunit of FRL-AP that make its PCB the most planar among AP variants. To determine the origin of the 25–30-nm red shift that occurs upon oligomerization, we performed biochemical manipulations on FRL-AP and additional spectroscopic characterization. We show that the spectroscopic properties of FRL-APs do not result from excitonic coupling between chromophores of adjacent protomers. We propose that the 25–30-nm red shift exhibited by AP, AP-B, and FRL-AP upon the transition from monomer to oligomer arises from a conformational change in the D-ring of the PCB chromophore on the α-subunit and the stabilization and tight binding of that chromophore that occurs upon oligomerization.

## Materials and methods

### Structural and sequence comparisons

Structures of AP, AP-B, FRL-AP, and PC were gathered from the protein data bank (PDB) under accession codes 4RMP, 4PO5, 8DDY, and 1KTP, respectively. Superpositions were performed with the software PyMOL (DeLano 2014) using the *super* command. Multiple sequence alignments were performed using Clustal Omega (Sievers et al. 2011). Surface electrostatics were calculated using the Adaptive Poisson-Boltzmann Solver (Jurrus et al. 2018).

### Site-specific mutagenesis and protein production

Site-specific variants of the ApcD4 and ApcB3 proteins were produced as described by (Soulier and Bryant 2021). The mutagenized genes were verified by DNA sequencing and were expressed in an *Escherichia coli* strain engineered to produce PCB and the bilin lyase CpcS as previously described (Soulier and Bryant 2021).

### Steady-state spectroscopy

FRL-AP (ApcD4-ApcB3) was prepared as described in (Gisriel et al. 2023). Absorption spectra were recorded on a Varian Cary 4000 UV–VIS spectrophotometer, emission spectra on a HORIBA Jobin–Yvon Fluorolog 3 fluorometer (at an OD < 0.05 cm^−1^) and circular dichroism (CD) spectra on a Chirascan CD spectrophotometer. To induce the disassociation of the FRL-AP sample, NaSCN was added until the 709-nm band disappeared. This occurred at a final concentration of 3.0-M NaSCN.

### CD/OD calculation

When two molecules are close by, an electronic coupling can occur between their excited states. The electronic coupling between an excited state of the first molecule *n* and that of the next molecule *m*, V_nm_, can be calculated according to the ideal dipole approximation (in units of cm^–1^):

1 $$V_{nm} = 5.04\frac{{\left| {\mu_m } \right|\left| {\mu_n } \right|\kappa }}{R^3 }$$

in which, $\left|{\mu }_{x}\right|$ is the length of the transition dipole moment for the excited state transition of molecule x in Debye and R is the center-to-center distance between the molecules m and n in nm. κ is the orientation factor and is calculated according to

2 $$\kappa = \hat{\mu }_m \cdot \hat{\mu }_n - 3(\hat{\mu }_m \cdot \hat{R})(\hat{\mu }_n \cdot \hat{R})$$

In which, ${\widehat{\mu }}_{x}$ is the unit vector for the transition dipole moment of the excited state transition of molecule x and $\widehat{R}$ is the unit vector for the line that connects the center of molecule m to that of n. When excitation energies of the two molecules are on the same order of magnitude, excitonic effects can occur. Once this happens, two new excitonic states replace the excited states of the individual molecules and these excitonic states become shared between the two molecules. To retrieve the energy levels of the exciton states, the exciton Hamiltonian H needs to be diagonalized. In H, the excitation energies of the isolated molecules (E_x_) are placed on the diagonal and the electronic coupling between them on the off-diagonal:

3 $$H = \left[ {\begin{array}{*{20}c} {E_m } & {V_{mn} } \\ {V_{mn} } & {E_n } \\ \end{array} } \right]$$

The eigenvalues of this matrix yield the energy levels of the exciton states J, E_J_, and the associated eigenvectors represent the wavefunction coefficients of the individual molecules to the particular exciton states C_Jx_.

The absorption signal (OD) of the exciton state J depends linearly on its associated dipole strength D_J_, whereas its D signal is linearly dependent on its associated rotational strength R_J_. The dipole strength of the exciton state D_J_ can be calculated according to the following formula:

4 $$D_J = \mathop \sum \limits_{m,n}^N C_{Jm} C_{Jn} \left( {\mu_m \cdot \mu_n } \right)$$

Its rotational strength R_J_ alternatively can be quantitatively expressed as follows:

5 $$R_J = - \frac{\pi }{2\lambda }\mathop \sum \limits_{m,n}^N C_{Jm} C_{Jn} (r_{mn} \cdot \mu_m \times \mu_n )$$

in which λ is the wavelength that corresponds to the energy of the exciton state $J\left( {\lambda = \frac{hc}{{E_J }}} \right)$ and r_*mn*_ is the distance vector connecting the centers of molecule *m* to *n*. These relations provide a handle to determine the presence and extent of excitonic effects in OD and CD spectra. In the case that the CD and OD spectra are purely of excitonic nature, then the following equation will hold:

6 $$\frac{CD}{{OD}} = \frac{4R_j }{{D_j }}$$

in which the ratio $\frac{CD}{OD}$ can be determined from the experimentally recorded spectra and the ratio $\frac{4{R}_{j}}{{D}_{j}}$ can be calculated based on a structural/physical model (Somsen et al. 1996). In the next paragraph, we describe how we calculated the ratio $\frac{4{R}_{j}}{{D}_{j}}$ for FRL-AP.

First, the exciton Hamiltonian needed to be established for FRL-AP. In the FRL-AP structure, most α-PCB chromophores are in the vicinity of a β-PCB and these α/β-PCB dimers are each in turn rather isolated from other PCB chromophores. Each dimer is moreover in a very similar configuration and environment (Gisriel et al. 2023). The exciton Hamiltonian can thus be established for a single α/β-PCB dimer. The electronic coupling in the α/β-dimers was calculated to be –96 cm^–1^ according to Eq. 1. For details about how the necessary parameters were retrieved, we refer the reader to (Gisriel et al. 2023). As an estimate of the exciton energies in FRL-AP, the wavelengths of the peaks of the bands in the red region of the absorption spectrum were taken. The higher-energy wavelength is 621 nm (16,103.1 cm^–1^) and the lower energy wavelength is 709 nm (14,104.4 cm^–1^). Equipped with the exciton energies and electronic couplings values, we worked back to establish the exciton Hamiltonian (Eq. 3). In this way, we determined the energy of the excited state of the individual α-PCB to be at 14,109.0 cm^–1^ (708.8 nm) and that of the β-PCB to be at 16,098.4 cm^–1^ (621.2 nm). This procedure also yielded the wavefunction coefficients C_Jx_. The theoretically predicted D_J_ and R_J_ values were consequently evaluated using Eqs. 4 and 5. Details about how the necessary parameters were derived are found in (Gisriel et al. 2023). In short, the transition dipole moment vectors for the PCB molecules were taken to be directed from the NA atom to the ND atom, their transition dipole moment magnitudes were set at 10 Debye, and their center-to-center distance was taken between the CHA atoms of the PCBs. The calculations were evaluated based on the cryo-EM structure of FRL-AP (Gisriel et al. 2023) (PDB 8DDY). A schematic overview of the involved vectors is presented in Supplementary Fig. 1.

## Results and discussion

### Origin of the spectral features of FRL-AP

**Fig. 1:**
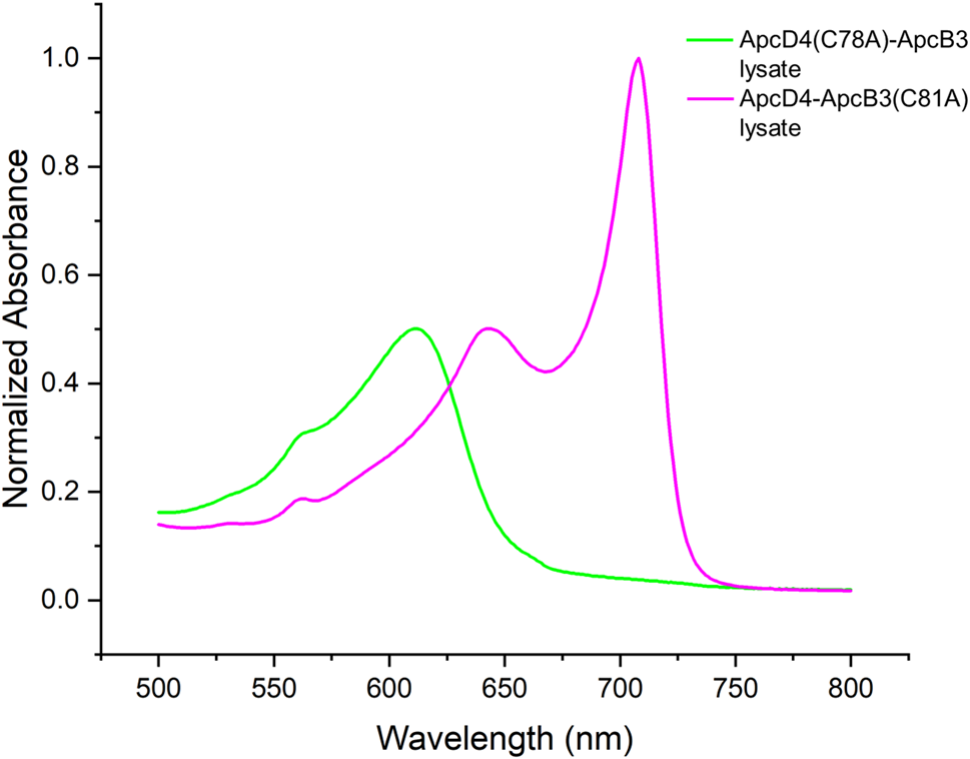
Comparison of variants of ApcD4-ApcB3 in which the PCB-ligating Cys residues were mutated to Ala. The spectra shown are from *E. coli* whole-cell lysates of strains producing the indicated proteins. To facilitate the comparison, the spectrum of recombinant ApcD4(C78A)-ApcB3 was normalized to the absorbance peak of ApcD4-ApcB3(C81A) at ~ 643 nm, which arises primarily from the noncovalently bound PCB chromophore of the β-subunit

To be certain which subunit was responsible for the two absorbance bands in FRL-AP, the chromophore-binding Cys residues of the α-subunit (ApcD4) and β-subunit (ApcB3) were mutated to Ala. The corresponding genes were co-expressed in an *E. coli* strain producing the appropriate partner wild-type subunit, PCB, and the CpcS lyase. The spectra of the cell lysates are recorded and are shown in Fig. 1. When the PCB-ligating Cys residue of the α-subunit is mutated to Ala, the resulting variant protein, ApcD4(C78A)-ApcB3, exhibited a single absorbance band with a maximum at ~ 615 nm. Evidently, ApcD4(C78A) is unable to bind and retain a PCB chromophore despite one still binding to ApcB3. The complex with ApcB3 could be purified by metal affinity chromatography due to the [His]10-tag on ApcD4(C78A) (data not shown). The spectrum of the lysate was nearly identical to the spectrum obtained when only the ApcB3 subunit was produced (Supplementary Fig. 2C). On the other hand, when the PCB-ligating Cys of the β-subunit (ApcB3) was mutated to Ala, the resulting variant protein, ApcD4-ApcB3(C81A), was more similar to the wild-type protein and exhibited two absorbance bands: a narrow one with its maximum at 709 nm and a broader one with a maximum at 643 nm. While the 709-nm band was identical to the band in the wild-type complex, the 643-nm band was red shifted by ~ 28 nm, as would be expected if the PCB chromophore was noncovalently bound by the β-subunit and retained one additional conjugated double bond. Thus, we conclude that, in the wild-type complex, the 709-nm absorbance band arises from the covalently bound PCB on the α-subunit and that the 617-nm absorbance band arises from the covalently bound PCB on the β-subunit.

### Overall subunit similarity

To gain insight into the similarities of FRL-AP subunits compared to those of AP and AP-B, we generated multiple sequence alignments (Supplementary Fig. 3), calculated the root-mean square deviation (RMSD) of the C_α_ superpositions of their structures (Table 1), and compared their absorbance spectra (Supplementary Fig. 2). The α-subunits of AP and AP-B are more similar to one another than either of these proteins is to the α-subunit of FRL-AP in both sequence identity and RMSD. The spectra of the three individual α-subunits (Supplementary Fig. 2B) differ substantially having maxima at 625, 650, and 680 nm for AP, AP-B, and FRL-AP, respectively. The trend is the same for the red-most maxima of the three oligomeric PBP complexes, which occur at 650, 670, and 709 nm, respectively, and which are mostly due to the absorbance of the PCB bound to the α-subunit (Soulier et al. 2022).

**Table 1:** Similarity of α-subunits and β-subunits

α-subunit RMSD (Å)	FRL-AP	AP	AP-B
FRL-AP	0.000	1.252	0.890
AP	1.252	0.000	0.645
AP-B	0.890	0.645	0.000

α-subunit identity (%)	FRL-AP	AP	AP-B
FRL-AP	100.0	41.67	45.22
AP	41.67	100.0	50.00
AP-B	45.22	50.00	100.0

β-subunit RMSD (Å)	FRL-AP	AP	AP-B
FRL-AP	0.000	0.518	0.570
AP	0.518	0.000	0.300
AP-B	0.570	0.300	0.000

β-subunit identity (%)	FRL-AP	AP	AP-B
FRL-AP	100.0	61.94	62.11
AP	61.94	100.0	87.10
AP-B	62.11	87.10	100.0

The table reports the RMSD of C_α_ atoms between subunits in units of Å and the sequence identity in units of %, for both α- and β-subunits from FRL-AP from *Thermostichus* sp. A1463 (Gisriel et al. 2023), AP from *Phormidium* sp. A09DM (Sonani et al. 2015), and AP-B from *Synechocystis* sp. PCC 6803 (Peng et al. 2014)

In any particular cyanobacterium, AP and AP-B share the same β-subunit (e.g., ApcB1 in both AP and AP-B in *Synechococcus* sp. PCC 7335). The small differences for the β-subunits of AP and AP-B in Table 1 are observed because the sequences are derived from different species, e.g., the β-subunits are both ApcB for *Phormidium* sp. A09DM and *Synechocystis* sp. PCC 6803 but share 87.10% identity (Peng et al. 2014; Sonani et al. 2015). When the β-subunits of AP or AP-B (both ApcB1) are superimposed onto the β-subunit of FRL-AP (ApcB3), they exhibit an RMSD of ~ 0.5 Å, which is quite small despite their sequence identity being only ~ 62%. Furthermore, there is essentially no difference in the absorbance contributions of the β-subunits, which in all cases are maximal at ~ 617 nm in oligomeric complexes and at ~ 615 nm for isolated subunits (Supplementary Fig. 2B and Supplementary Fig. 2C). Thus, the absorbance spectral differences of oligomeric AP, AP-B, and FRL-AP are mostly due to the properties of the α-subunits and not the β-subunits. A substantial narrowing of the red-most absorbance bands of the α-subunits upon oligomerization is also observed (Supplementary Fig. 2A and Supplementary Fig. 2B), which suggests that the α-PCBs are more rigidly bound in the oligomers than they are in the isolated subunits. This is reflected by a very small Stokes shift (5 nm) in the fluorescence emission of FRL-AP (Gisriel et al. 2023).

### Comparison of chromophore environments

**Fig. 2:**
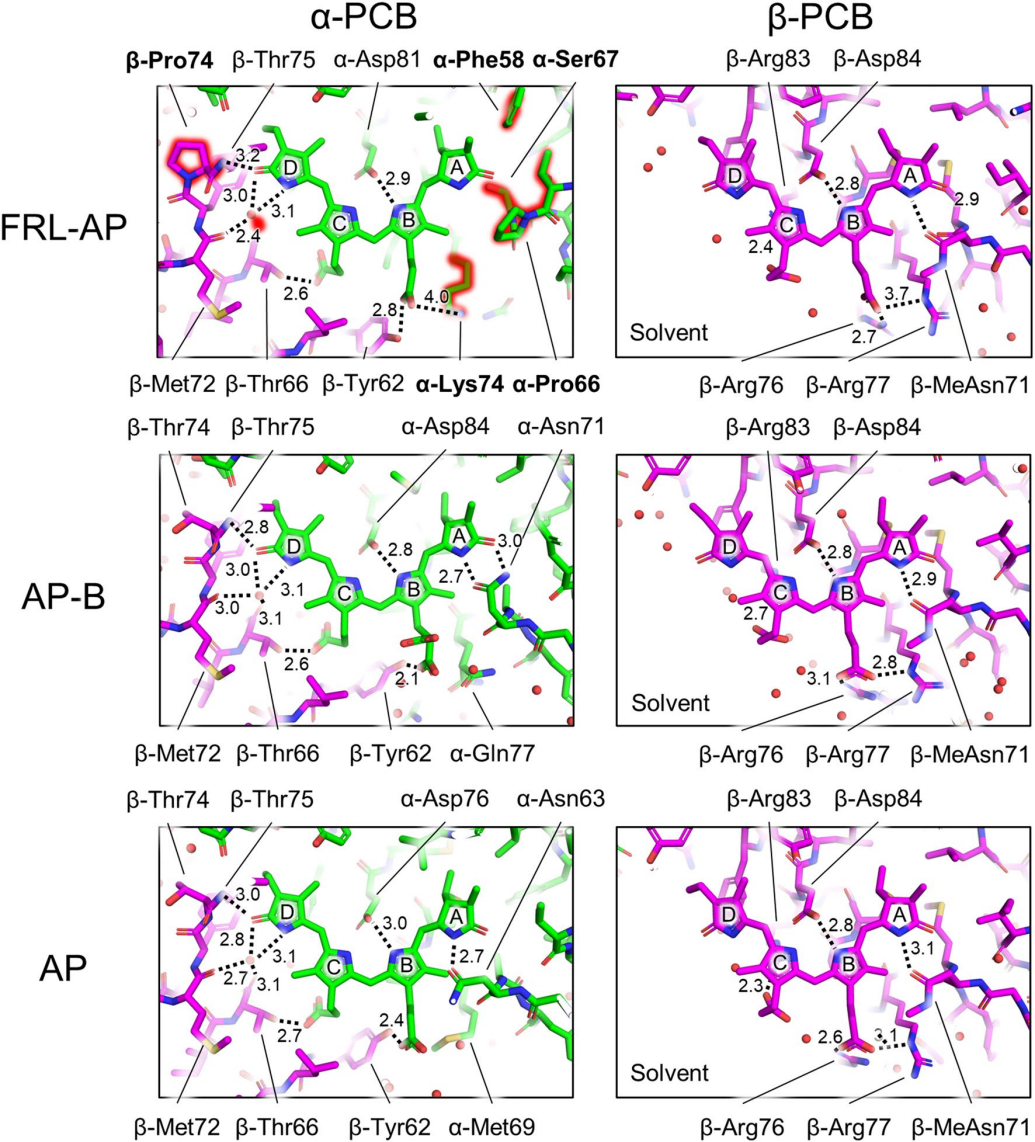
Comparison of protein residues near chromophores in the α-subunits and β-subunits of FRL-AP, AP, and AP-B. The residues near the chromophores on the α- (left column) and β-subunits (right column) are shown in stick representation for FRL-AP (top row), AP-B (middle row), and AP (bottom row). H-bonds are denoted by dashed lines and the corresponding distance measurement is labeled in units of Å. In the panel for the chromophore on the α-subunit of FRL-AP (top left), major differences compared to AP-B and AP are highlighted in red glow and non-conserved residues have bold labels. For the chromophores on the β-subunits, the solvent-exposed region is labeled. Note that in PBS structures containing AP and AP-B that assemble as toroids, the (apparently) solvent-exposed region would be occupied by a linker protein

In solution, PCB, the bilin chromophore bound by PC and by all AP variants, exhibits a cyclo-helical *ZZZ syn-syn-syn* geometry (Mizutani and Yagi 2004), which has not been observed in most PBP. Instead, the bilins in nearly all PBPs exhibit *ZZZ anti-syn-anti* geometry. The only exception is the bilin-binding domain of the core-membrane linker PBP (ApcE), which binds a PCB chromophore in *ZZZ syn-syn-anti* geometry, the same configuration found in phytochromes (Tang et al. 2015). Therefore, the PCB absorbance is effectively tuned by the protein environment that stabilizes stretched and extended configurations of the bilins. The bilin structures for the β-subunits of AP, AP-B, and FRL-AP are relatively similar, while this is not the case for the α-subunits (Gisriel et al. 2023). In particular, the pyrrole ring A of the PCB chromophore of the FRL-AP α-subunit is more planar and parallel to the plane of the other rings than the PCBs bound to the α-subunits of AP and AP-B (Gisriel et al. 2023). This finding extends the structure-based conclusions of Peng et al. (2014) for AP and AP-B, who correlated PCB coplanarity with lower energy absorbance, to include FRL-AP. Additionally, it is consistent with the conclusions based on homology modeling and site-specific mutagenesis that supported the same conclusion (Soulier and Bryant 2021). We therefore compared the detailed protein environments of FRL-AP with AP and AP-B to investigate the cause(s) of the observed structural differences (or lack thereof) among chromophores (Fig. 2).

The residues surrounding the PCB chromophore of the β-subunit are essentially identical among FRL-AP, AP-B, and AP and comprise only residues derived from the β-subunit to which a given β-chromophore is covalently bound. This is consistent with (a) the relatively high similarity among β-subunits in both sequence and structure (Table 1) and (b) the similar variance among their chromophores (Gisriel et al. 2023) that give rise to very similar absorption contributions (Supplementary Fig. 2C). Furthermore, this is consistent with findings of Soulier and Bryant (2021), who found that it was possible to form chimeric proteins with FRL absorbance when ApcD4 was combined with either ApcB1 or ApcB3 subunits from the same *Thermostichus* sp. strain. The α-chromophore of FRL-AP, on the other hand, has unique interactions and surrounding residues that are not found in AP-B or AP (Fig. 2), which is consistent with the differences in the absorbance maxima of the three α-subunits (Supplementary Fig. 2). In all PBPs, the environment of the α-chromophore comprises residues from the BE loop in the α-subunit to which it is bound, in the vicinity of rings A and B of PCB, and also the most N-terminal residues of helix E in the β-subunit from the adjacent protomer, in the vicinity of pyrrole rings C and D (see Supplementary Fig. 4 for helix nomenclature as described previously (Schirmer et al. 1986)). The following features of the α-chromophore environment in FRL-AP differ in AP-B or AP: (a) pyrrole ring A does not exhibit obvious H-bonding in FRL-AP but does exhibit H-bonding in AP and AP-B; (b) a bulky Phe sidechain, α-Phe58, is present near ring A in FRL-AP, while Leu residues occur in AP and AP-B; (c) the carbonyl moiety of ring D accepts an H-bond from the backbone nitrogen atom of β-Thr75 rather than β-Thr74 of helix E, which decreases the extent of the H-bonding network around pyrrole ring D relative to that observed in AP and AP-B; and (d) the propionate moiety on ring B is charge compensated by an interaction with an α-Lys sidechain in helix E that is conserved in FRL-AP α-subunits but does not occur in the α-subunits of AP or AP-B.

**Fig. 3:**
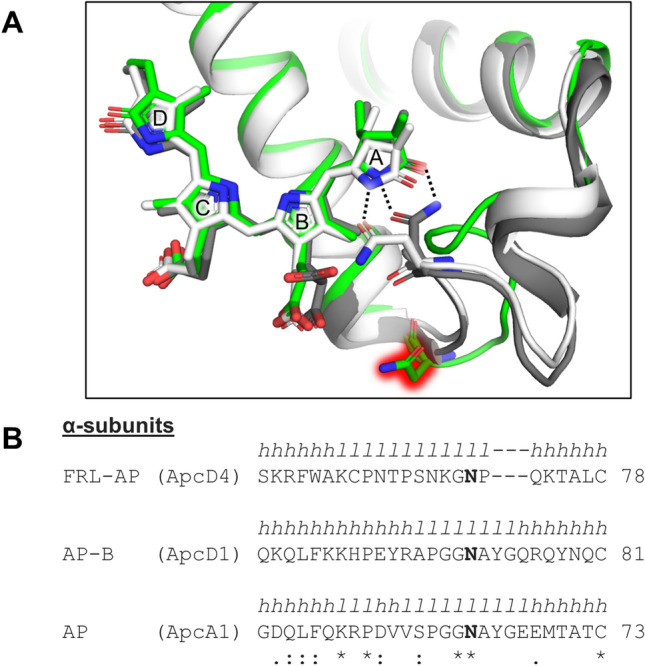
Unique ApcD4 looping region between helices B and E near the A- and B-rings of the α-chromophore in FRL-AP. **A** Structural superposition showing only α-subunits from FRL-AP (green), AP-B (gray), and AP (white). The α-chromophore is shown in stick representation. The Asn sidechain that participates in H-bonding in AP and AP-B (denoted by dashed lines), but does not in FRL-AP, is shown in stick representation, where the latter is highlighted in red glow. **B** Partial sequence alignment of the region in panel (A) where the Asn residue is bold. The secondary structure at each position is shown above the sequence where *h* corresponds to an α-helix and *l* corresponds to a loop. The Clustal Omega sequence conservation identifier is shown below the alignment

The lack of H-bonding to pyrrole ring A and the propionate charge compensation in FRL-AP are due to the non-conserved looping region that is also structurally unique compared to AP and AP-B (Fig. 3A). In AP and AP-B, a conserved Asn sidechain in the BE loop of the α-subunit provides H-bonding interactions to the respective A rings, which must contribute to stabilizing their conformations. Although this Asn is conserved in the α-subunit sequence of FRL-AP, the BE loop is substantially different, containing a deletion and several non-conserved residues (Fig. 3B). As a result, the conserved Asn in FRL-AP is instead found as a surface residue, ~ 12 Å away from the α-chromophore. Thus, unlike the α-chromophores of AP and AP-B, pyrrole ring A is not stabilized by H-bonding from α-Asn71 in FRL-AP, which is interesting because H-bonding interactions are frequently observed to red shift the absorbance of cyclic tetrapyrroles (e.g., (Llansola-Portoles et al. 2020)). Residue α-Asn71 in FRL-AP is therefore conserved in sequence but not structure or function. This absence of H-bonding is consistent with the lack of absorbance changes upon the mutation of possible H-bond donors near the α-chromophore in FRL-AP (including α-Asn71) reported recently (Soulier and Bryant 2021). The loop deletion that causes the relocation of α-Asn71 is conserved in the sequences of all other FRL-AP α-subunits (i.e., ApcD2, ApcD3, and ApcD5). Therefore, this modified loop appears to be a hallmark feature of all members of the AP superfamily that absorb FRL and not just of ApcD4-ApcB3 (also see Soulier et al. 2020 and Soulier and Bryant 2021).

Stabilization of pyrrole ring A in FRL-AP may instead be conferred by cooperative steric interactions from the non-conserved loop itself; it probably exhibits a more rigid conformation due to its shorter length (Fig. 3). Additionally, α-Phe58 is also likely to stabilize the A-ring of the α-chromophore due to its bulkier sidechain compared to the Leu residue found in AP and AP-B (Fig. 2). This Phe sidechain is only ~ 3.5 Å from ring A and is conserved among all FRL-AP sequences, so it too appears to be a hallmark of members of the AP family that absorb FRL, not just ApcD4-ApcB3. Thus, we propose that the FRL-AP-specific looping region and nearby Phe sidechain causes a nearly planar conformation of ring A of the α-chromophore due to steric stabilization in AP family members that absorb FRL, contributing to the bathochromic shift of their absorbance maxima. This is consistent with (a) the observation that mutation of residues in this loop to bulkier sidechains frequently blue-shifted the red-most absorbance maximum, probably by disrupting the important cooperative steric stabilization of the BE loop on the α-chromophore (Soulier and Bryant 2021); (b) the observation that mutation of α-Phe58 to Ala in ApcD4-ApcB3 causes a decrease in the 709-nm peak intensity; and (c) the small Stokes shift in the fluorescence emission for the protein (Soulier and Bryant 2021; Gisriel et al. 2023). Notably, steric interactions were also suggested to play a role in red shifting the α-chromophore in AP-B (Peng et al. 2014).

Pyrrole rings C and D of the α-chromophore partially define the protomer–protomer interface, which is thought to be more stabilized in AP variants compared to PC (Peng et al. 2014). In AP and AP-B, the carbonyl moiety of pyrrole ring D of the α-PCB accepts an H-bond from the backbone nitrogen atom of β-Thr74, which is the most N-terminal residue in helix E and a nearby water molecule (Fig. 2). In FRL-AP, position 74 of the β-subunit is instead a Pro residue that brings the backbone amide nitrogen atom of the subsequent residue, β-Thr75, in proximity to serve as an H-bond donor to the carbonyl oxygen atom of pyrrole ring D (Fig. 2). Consequently, the H-bond appears weaker in FRL-AP, with a distance of 3.2 Å compared to 2.8 Å and 3.0 Å in AP-B and AP, respectively. The other H-bond donor to the carbonyl oxygen of pyrrole ring D of the α-PCB is a water that similarly occurs in FRL-AP. In AP and AP-B, the water is within H-bonding distance of three other atoms that could be involved in H-bonding: (i) the pyrrole nitrogen atom of ring D; (ii) the backbone carbonyl oxygen atom of β-Met72; and (iii) the backbone carbonyl moiety of β-Thr66. In FRL-AP, the former two maintain H-bonding distances to that water, but the backbone carbonyl moiety of β-Thr66 is not within H-bonding range of the water molecule. These differences in FRL-AP compared to AP and AP-B do not appear to substantially influence the protein environment near ring D of the α-chromophore, but we note that the nearby, non-conserved β-Pro74 residue may additionally confer rigidity, possibly stabilizing the α-chromophore in FRL-AP which could also contribute to the small Stokes shift observed for its fluorescence emission. However, β-Pro74 is only conserved in ApcB3 sequences; therefore, this configuration is likely to be quite specific to the ApcD4-ApcB3 complex. It may be that the slightly different environment near this interface contributes to the helical oligomerization in FRL-AP (ApcD4-ApcB3).

Residue α-Lys74, whose sidechain acts as a counter ion to the propionate moiety of pyrrole ring B of the α-PCB of FRL-AP, is not conserved in the structures of AP or AP-B (Fig. 2); instead, Met and Gln are found at those positions, respectively (Supplementary Fig. 3). Indeed, the alignment of multiple AP and AP-B sequences shows that none of the ApcA1 or ApcD1 sequences conserve this residue; however, it is also not entirely conserved among the α-subunit sequences of FRL-AP: it is conserved in ApcD4, ApcD3, and ApcD5, but not ApcD2 (Soulier et al. 2020). This difference could be significant, because ApcD3 and ApcD5 produce proteins that strongly absorb FRL when combined with ApcB2 (Soulier et al. 2020). In ApcD2, Tyr or Phe is found at this position, and correspondingly, this subunit produced a protein with the weakest FRL absorbance when combined with its cognate β-subunit, ApcB2 (Soulier et al. 2020). This suggests that α-Lys74 does not contribute to the absorption of FRL directly but may contribute to maintaining the unique structure of the BE loop. This is supported by the observation that mutation of α-Lys74 to Ala had little influence on the absorbance spectrum (Soulier and Bryant 2021). Furthermore, charge compensation of the propionate moiety would not be expected to influence the extended conjugation of the pyrrole rings that would influence the absorbance spectrum of the chromophore. However, its presence raises the question of whether the local electrostatics could influence the spectral characteristics of the chromophores.

**Fig. 4:**
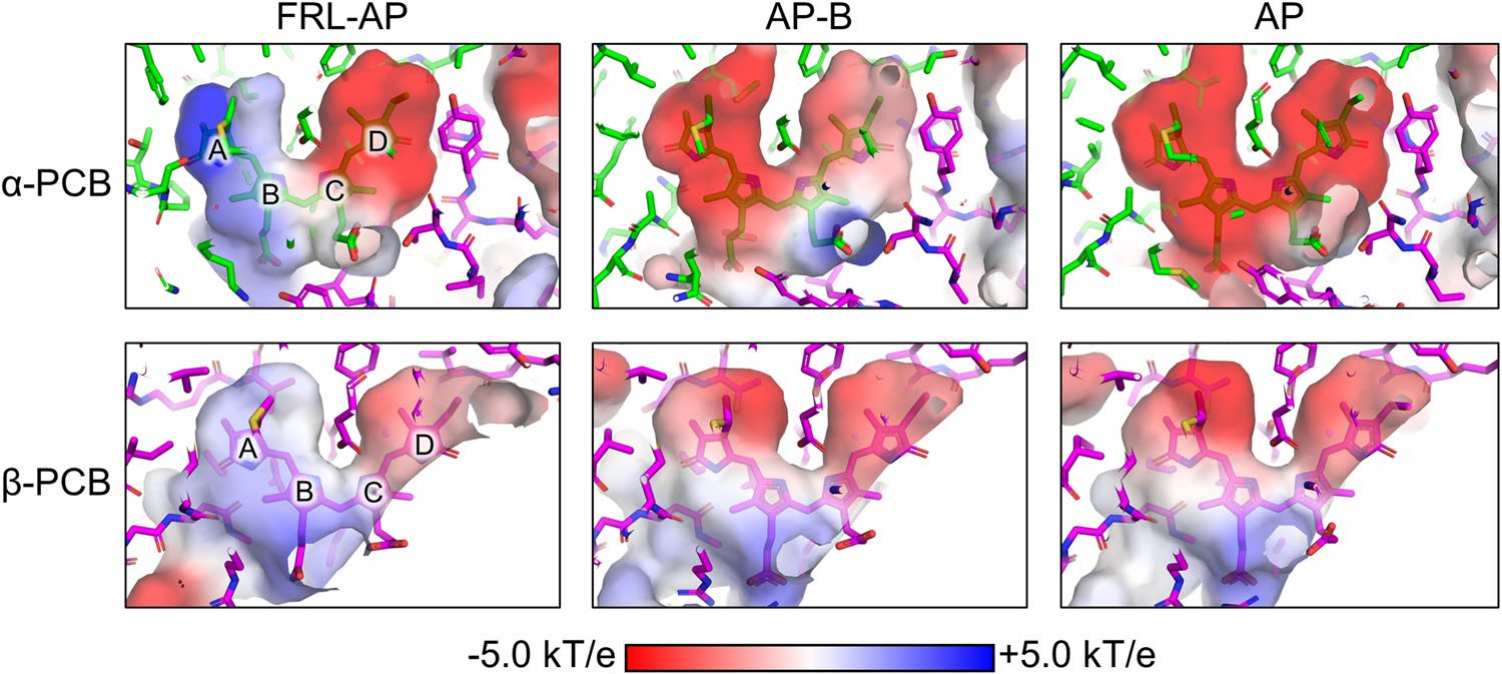
Surface electrostatic potentials of the chromophore-binding cavities in FRL-AP, AP-B, and AP. The surface electrostatic potential maps of the α-chromophore (top row) and β-chromophore (bottom row)-binding pockets are shown for FRL-AP (left column), AP-B (center column), and AP (right column). The scale bar is shown below the panels. The pyrrole rings of PCB are labeled in the panels for the α-chromophore (top left) and β-chromophore (bottom left) of FRL-AP

To investigate possible differences in the electrostatic environments of the chromophores in FRL-AP, AP, and AP-B, we calculated the electrostatic surface potentials of the empty bilin-binding pockets (Fig. 4). Whereas the binding pockets for the β-PCB in all three proteins exhibit similar surface electrostatic potentials, the binding pockets for the α-PCBs exhibit much greater differences (Fig. 4). AP and AP-B both exhibit negatively charged environments near pyrrole rings A and B, whereas the corresponding region in FRL-AP is substantially positively charged, especially near ring A. An analysis of the FRL-AP structure suggests that this region, which comprises the non-conserved BE loop (Fig. 3), has the backbone amide nitrogen atoms of α-Pro66 and α-Ser67 directed toward it (Fig. 2), which is the most likely cause of the positive charge. It should be noted that the mutation of these two residues to Ala did not substantially alter the absorbance spectrum (Soulier and Bryant 2021), which is reasonable if the backbone amide nitrogen atoms interact with the α-chromophore rather than their sidechains. The protein environments surrounding the C and D pyrrole rings of the α-chromophore in the surface electrostatic potential maps for all three proteins are primarily negatively charged, but to a lesser extent in AP-B. Thus, the non-conserved α-subunit loop near rings A and B likely provide steric stabilization of the α-chromophore but additionally appear to create a more positively charged environment, which could contribute to the bathochromic shift of the absorbance spectra observed for oligomeric FRL-AP and its isolated α-subunit (Supplementary Fig. 2).

### Assessment of excitonic effects

The availability of structural and spectroscopic data for FRL-AP provides an opportunity to address whether excitonic effects contribute to the red shift of FRL-AP upon oligomerization and to address the current view of such excitonic effects among AP variants in general. The presence and/or extent of excitonic coupling in AP variants have been a longstanding debate (e.g., see MacColl et al. 2003; MacColl 2004). The main experimental results supporting the presence of excitonic interactions are as follows: (1) upon oligomerization of the (α/β)-protomers, the absorption spectrum acquires a narrow red-shifted absorbance band, which some have interpreted as the result of excitonic energy splitting and band narrowing due to the electronic coupling between α- and β-chromophores from adjacent protomers (MacColl 2004); (2) the CD spectrum of trimeric AP consists of several negative and positive bands in the red region of the spectrum that are not observed in AP (α/β) monomers, which were interpreted to be excitonic in nature (Csatorday et al. 1984); and (3) an ultrafast energy transfer component of 10–30 fs and an initial anisotropy of 0.58–0.70 were observed in oligomerized AP, which would be inconsistent with Förster-type energy transfer and indicative of internal conversion between exciton levels (Edington et al. 1995). The presence of strong excitonic effects are, however, seemingly at odds with the large energy gap of ~ 750 cm^–1^ between the main transitions corresponding to the β and α chromophores with respect to their electronic coupling (~ 110–220 cm^–1^) (Csatorday et al. 1984; Edington et al. 1996; Womick and Moran 2009). More recently, it has been suggested that excitonic interactions in AP are not formed between the two main transitions of the α- and β-PCB chromophores, but instead result from a coupling between the main transition of the β-PCB and a vibronic mode of the α-PCB, which can satisfactorily explain the ultrafast relaxation processes observed in AP (Womick and Moran 2009, 2011). These conflicting views led us to assess the possible presence and extent of excitonic interactions in FRL-AP and to relate the results to prior studies of AP and AP-B.

**Fig. 5:**
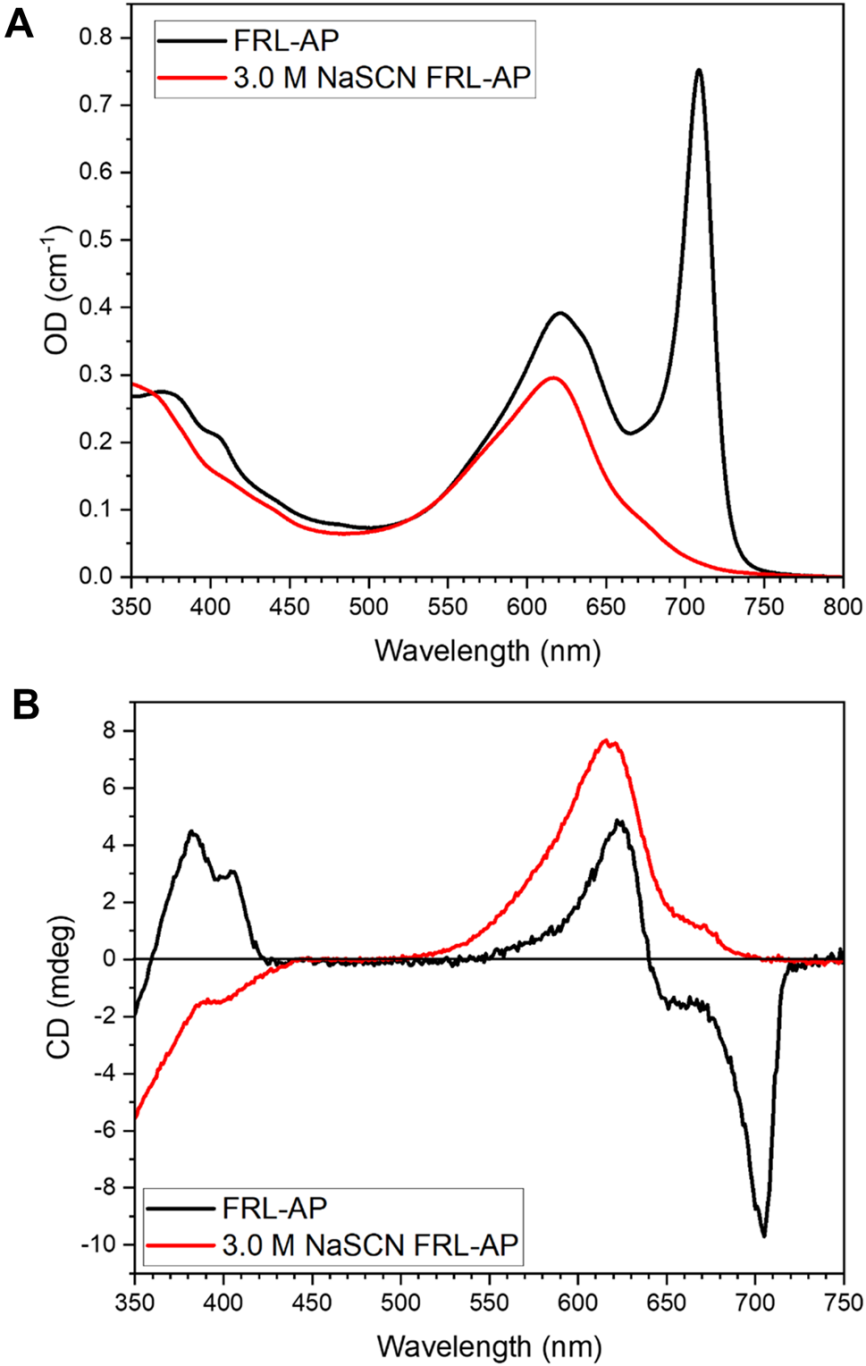
Absorption (**A**) and CD (**B**) spectra of FRL-AP (black) and of its subunits (red) as induced by the addition of 3.0-M NaSCN. The spectra all correspond to the same amount of sample

To assess the presence and extent of excitonic effects in FRL-AP, we recorded and compared its absorption and CD spectra (Fig. 5). The CD spectrum of FRL-AP (Fig. 5B) shows positive signals in the red portion of the spectrum in the region of the β-PCB absorption and negative signals in the region of the α-PCB, which could be indicative of excitonic interactions. The extent of excitonic interactions in a system can be assessed by comparing the amplitude of the CD signal with respect to the absorption (OD) signal to the theoretically expected ratio between the rotational (R_j_) and dipole (D_j_) strength. If the CD spectrum were purely excitonic, the relation $\frac{CD}{OD}=\frac{4{R}_{j}}{{D}_{j}}$ holds (Somsen et al. 1996) (see Materials and Methods). The calculation of the excitonic rotational strength for the high energy band gives a positive value and thus the low energy band must have a negative value, which is opposite to the observed pattern in the experimental CD spectrum. Moreover, for the high energy band, a ratio $\frac{4{R}_{j}}{{D}_{j}}$ of –7.3 × 10^–5^ is computed, while the experimentally determined $\frac{CD}{OD}$ ratio is five times larger: 3.8 × 10^–4^. This is also the case for the low energy band: $\frac{4{R}_{j}}{{D}_{j}}$ is 5.8 × 10^–5^, while $\frac{CD}{OD}$ is –3.9 × 10^–4^. This analysis therefore convincingly nullifies the hypothesis that the CD spectrum of FRL-AP has an excitonic origin.

Because excitonic effects can be excluded, the CD spectrum should be the sum of the intrinsic CD signals of the α-PCB and β-PCB chromophores. To estimate the magnitude of the intrinsic CD signals of the PCBs in FRL-AP, we induced the dissociation of the FRL-AP into protomers (αβ) by adding the chaotrope 3.0-M NaSCN, which was the minimal amount of chaotrope required to eliminate completely the absorbance band at 709 nm. The absorption and CD spectra of the resulting solution are shown in Fig. 5. The absorption spectrum lacks the characteristic 709-nm band of oligomeric FRL-AP, which indicates that the dissociation to protomers and possibly some individual subunits is complete with 3.0-M NaSCN. Additionally, the absorption spectrum after dissociation shows a peak at 616 nm, which nicely corresponds to the isolated β-PCB subunit absorption (Soulier et al. 2020; Soulier and Bryant 2021), and a shoulder around 678 nm, which can be attributed to the α-PCB chromophore (Soulier et al. 2020; Soulier and Bryant 2021). The fluorescence emission spectrum shows contributions from both β-PCB (~ 640 nm) and α-PCB (~ 700 nm) and is similar in shape to spectra of protomers of AP and AP-B. This spectrum shows that the two subunits are not efficiently connected and are thus far apart, as expected for protomers and isolated subunits (Supplementary Fig. 5). The red region in the CD spectrum shows the same shape as the absorption spectrum and notably the amplitude of the CD is on the same order of magnitude as the CD of the FRL-AP (Fig. 5B). This is a strong indication that the CD spectrum of FRL-AP can predominantly be attributed to intrinsic factors and not to excitonic coupling.

The CD spectrum of FRL-AP is, however, purely positive in the red region that originates from the PCB of the β-subunit, whereas the CD spectrum of the FRL region representing the contribution of the α-PCB is negative. What is the origin of this difference? In this respect, it is interesting to compare our results to the case of AP-B. The absorption spectrum of AP-B is similar in shape to the FRL-AP spectrum, except that the red-most band is maximal at 670 nm instead of 709 nm as in FRL-AP (Glazer and Bryant 1975; Peng et al. 2014). Moreover, the CD signal of AP-B is very similar in shape to that of FRL-AP and its relative amplitude compared to the OD spectrum is also comparable to that of FRL-AP. However, the CD signal of AP-B (αβ)-protomers shows the opposite signs for the α-PCB and β-PCB (−/+ instead of +/−) (Peng et al. 2014). As excitonic interactions can be excluded in (αβ)-protomers due to the large separation between the chromophores (MacColl 2004; Gisriel et al. 2023), these results show that the individual PCB chromophores can undergo CD sign switches. This has been observed in studies of PCBs in phytochromes and cyanobacteriochromes, in which sign flips of the CD signal can be observed upon photo-activation (Rockwell et al. 2009). It has been argued that the sign of the CD signal of PCBs in phytochromes depends on the conformation of the D-ring (Rockwell et al. 2009). By analogy, it is possible that conformational changes at the α-PCB D-ring are responsible for the differences in CD signal between (αβ)-protomers and oligomers in FRL-AP.

In the absorption spectra (Fig. 5A) it is interesting to note that the oscillator strength of the α-PCB is greatly decreased by the dissociation to protomers and subunits. Moreover, the emission spectrum of the subunits (Supplementary Fig. 5) reveals large Stokes shifts for both the β- and α-PCBs (see (Soulier et al. 2020)). These observations exemplify the hypersensitivity of bilin chromophores to their environment and support the hypothesis that upon oligomerization, the conformational flexibility of the α-PCB decreases significantly, which leads to a drastic narrowing and amplitude increase of its absorption band.

### Origin of the 30-nm red shift upon oligomerization

The absorbance maxima of all AP variants—AP, AP-B, and FRL-AP—exhibit a bathochromic shift of ~ 25 to 30 nm and band narrowing upon the transition from the (α/β) protomer to oligomer, (α/β)_3_ or (α/β)_*n*_. The isolated α-subunits of AP, AP-B, and FRL-AP have broad, largely featureless absorbance bands that absorb maximally at 625 nm, 650 nm, and 680 nm, respectively (Supplementary Fig. 2B). The absorbance spectra of the β-subunits, ApcB1 and ApcB3, are very similar to one another and absorb maximally at ~ 615 nm (Supplementary Fig. 2C). Furthermore, the spectra of the β-subunits resemble the higher-energy absorbance band of the oligomeric proteins (Supplementary Fig. 2A). Thus, it is clear that the PCB chromophore of the α-subunit must undergo a very substantial change upon the transition from monomer to oligomer, while the β-subunits remain largely unaffected by oligomerization.

**Fig. 6:**
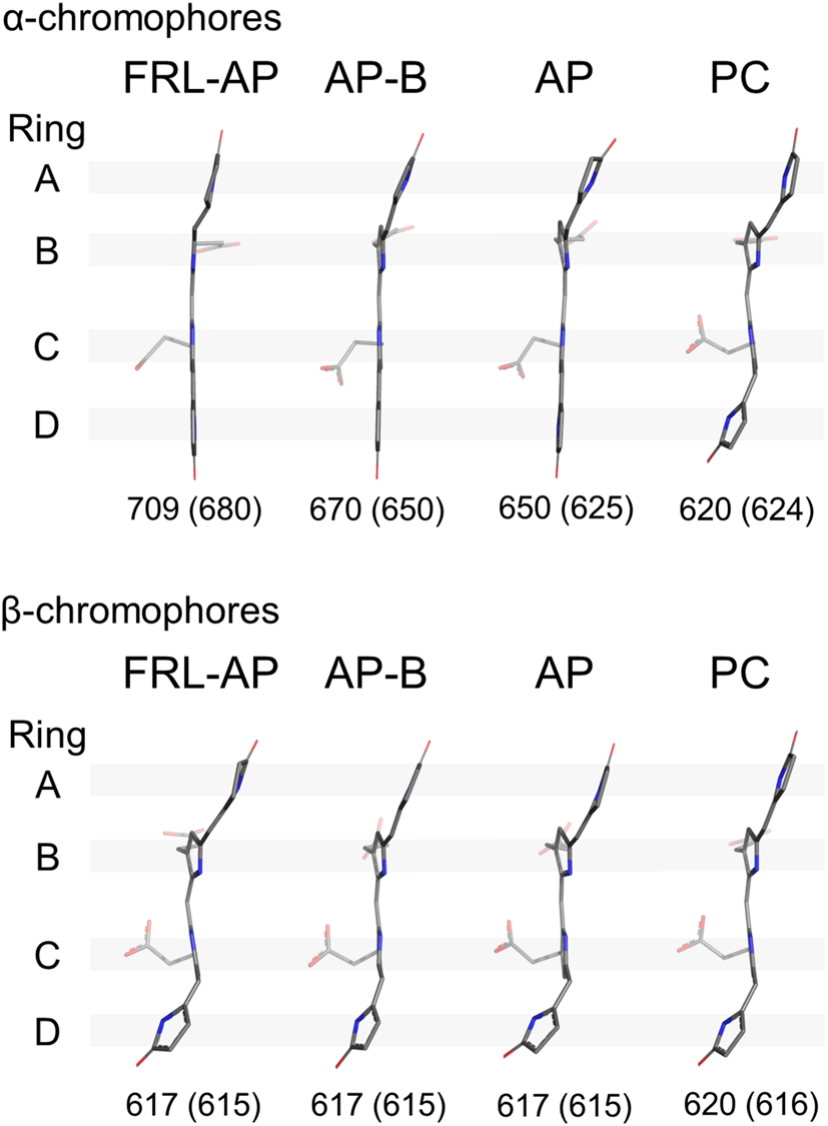
Comparison of PCB chromophores in selected PBPs. The chromophore structures are from the α- and β-subunits of oligomeric PBPs: FRL-AP, AP-B, AP, and PC. These correspond to PDB entries 8DDY, 4PO5, 4RMP, and 1KTP, respectively. Below each chromophore is the absorbance maximum contribution of the chromophore in the oligomeric state and in parentheses the absorbance maximum of the chromophore in the isolated subunit, which should be similar to the value for protomers. The absorbance maxima shown for PC were taken from Debreczeny et al. (1993, 1995)

Figure 6 shows a comparison of the PCB chromophore conformations for the α- and β-subunits of AP, AP-B, and FRL-AP as found in oligomers. The chromophores are shown in an edge view to visualize and accentuate the degree of coplanarity of the pyrrole rings of the PCBs. Also included are the PCB chromophores attached to Cys82 and Cys84 of the α- and β-subunits, respectively, of a hexameric cyanobacterial PC (Adir et al. 2002). Visual inspection of the structures reveals that they fall into two easily discernable groups. In the first group, which includes the α-subunits of AP, AP-B, and FRL-AP in oligomers, pyrrole rings B, C, and D are coplanar (FRL-AP) or nearly so (AP and AP-B). The proteins binding these chromophores have absorbance maxima at 650, 670, and 709 nm. As previously reported by Gisriel et al. (2023), the pyrrole ring A moiety of FRL-AP is more coplanar and less twisted relative to ring B than is the case for the PCBs of the AP and AP-B α-subunits. In the second group, the PCB chromophores are clearly twisted and their pyrrole rings are much less coplanar overall. Importantly, all PCB chromophores that have similar twisted conformations have similar spectra with absorbance maxima between 615 and 625 nm. This group includes the β-subunits of the three AP variants as well as two of the three PCB chromophores of a cyanobacterial PC. Unfortunately, all structures shown in Fig. 6 were obtained from oligomeric proteins—helical nanotubes for FRL-AP, trimers for AP and AP-B, and hexamers for PC—because no structures exist for protomers of these proteins.

The isolated α- and β-subunits of AP and the α- and β-subunits of PC have very similar absorbance spectra, with maxima between 615 and 625 nm (Supplementary Fig. 2). Moreover, these spectra are very similar to those of monomeric (i.e., protomeric) AP and AP-B (MacColl et al. 2003; Peng et al. 2014). If the spectra are the same, then it must be the case that the chromophores have very similar structures as well. Thus, we infer that the chromophores in isolated α-subunits as well as protomers of AP must have the twisted conformation of the D-ring that is retained by the β-subunit of AP after oligomerization (Fig. 6). However, the PCB chromophore on the α-subunit must therefore change from the twisted to the coplanar conformation upon oligomerization. Thus, we propose that this change is responsible for the characteristic narrow absorbance band of AP at 650 nm.

**Fig. 7:**
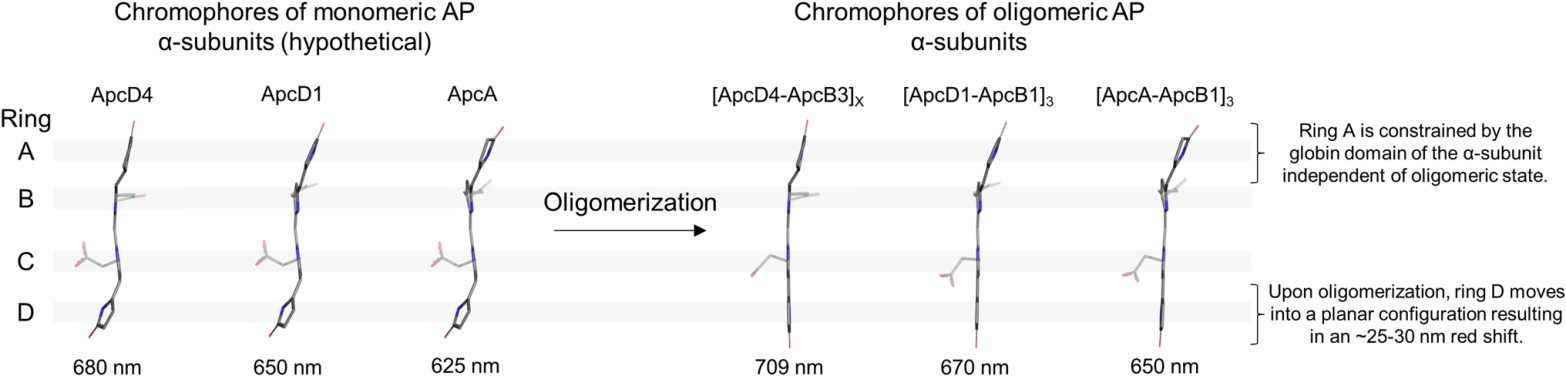
Hypothetical structures of chromophores in protomeric (monomeric) FRL-AP, AP-B, and AP α-subunits and alteration of rings C and D upon oligomerization. On the left, the hypothetical structures of the chromophores bound in protomeric ApcD4 (α-subunit of FRL-AP), ApcD1 (α-subunit of AP-B), and ApcA (α-subunit of AP) were constructed using rings A and B from their structures (8DDY, 4PO5, and 4RMP, respectively), and rings C and D from the structure of the α-chromophore from PC (1KTP). The structures of oligomeric FRL-AP, AP-B, and AP on the right are the coordinates derived experimentally (PDB entries 8DDY, 4PO5, and 4RMP, respectively)

The protein environments of the D-rings of the α-PCB chromophores are very similar among AP variants, and they lie at the interface between (α/β)-protomers upon oligomerization. As described above, we and others have excluded excitonic effects as an explanation for the ~ 25–30-nm red-shift and band narrowing that occurs upon oligomerization. Furthermore, electrostatic effects also seem unlikely to account for most of these effects. Therefore, we propose that the spectral changes that occur upon oligomerization of FRL-AP arise from a conformational change of the C- and D-rings of the α-PCB chromophore (Fig. 7). Although no structure of any (α/β) protomer exists for any AP variant to test this hypothesis directly, it is nevertheless relatively clear that in the absence of the adjacent β-subunit, the D-ring of the α-PCB would be less constrained and probably more exposed to solvent. One could therefore imagine that a rotation of the D-ring into its final planar configuration might occur during oligomerization when the β-subunit of a second protomer binds near the PCB on an α-subunit. This binding could lead to a tightening of the binding pocket to exclude solvent access and increase rigidity of the planar chromophore. These changes would lead to the band narrowing and bathochromic shift of the corresponding absorbance band, leading to a decrease in the Stokes shift, increased oscillator strength, and changes in the rotational strength/CD signal. Importantly, as shown in Fig. 7, this mechanism would be ubiquitous among AP variants, explaining why a similar red shift is observed in AP, AP-B, and FRL-AP. Note that this explanation does not contradict, but rather supplements, the current picture related to the vibronic excitons in AP: the vibronic excitonic interactions occur in the spectral region between the absorption peaks of the β- and α-PCB, which thus do not explain the red shift and band narrowing of the main transition of the α-PCB (Womick and Moran 2011).

## Conclusion

The differences in the spectra of isolated α-subunits of AP variants are primarily due to the protein environments near their pyrrole A rings, which result in spectra with maxima at 625, 650, and 709 nm for AP, AP-B, and FRL-AP, respectively. For FRL-AP, we suggest that the electrostatic environment near this ring might also play a role in the significantly red-shifted spectrum. The isolated α-subunits and protomers of AP variants probably have an unconstrained and twisted ring D, most likely being positioned out of the plane with the rest of the chromophore and exhibiting a higher energy relative to the oligomeric state. Upon oligomerization, ring D becomes constrained to become more coplanar with the rest of the chromophore, causing the ~ 25–30-nm red shift that occurs in all AP variants. On the other hand, the conformation of the PCB on the β-subunit is not significantly influenced by oligomerization nor are excitonic effects of the α- and β-PCBs an important contributing factor in the observed spectra of oligomers of AP variants. These results therefore provide a molecular mechanism linking the structures of AP variants to their corresponding spectral data that extends our understanding of AP variants to include FRL-APs.

## Supplementary Information

Below is the link to the electronic supplementary material.

Supplementary file1 (DOCX 807 kb)
