# Supplementary material for: Structural comparison of allophycocyanin variants reveals the molecular basis for their spectral differences
Source: Photosynth Res. 2023 Sep 29;162(2-3):157–70. doi: 10.1007/s11120-023-01048-4 (PMC11614940; doi:10.1007/s11120-023-01048-4)
Supplement: Supplementary file 1 — Supplementary file1 (DOCX 807 kb) [file 11120_2023_1048_MOESM1_ESM.docx]

**Supporting Information for**

**Structural comparison of allophycocyanin variants reveals the molecular basis for their spectral differences**

Christopher J. Gisriel^1,†^, Eduard Elias^2,†^, Gaozhong Shen^3^, Nathan T. Soulier^3,‡^, Gary W. Brudvig^1,4^, Roberta Croce^2,^*, and Donald A. Bryant^3,^*

^1^Department of Chemistry, Yale University, New Haven, CT 06520, USA.

^2^Department of Physics and Astronomy and Institute for Lasers, Life and Biophotonics, Faculty of Sciences, VU University Amsterdam, 1081 HV Amsterdam, Netherlands

^3^Department of Biochemistry and Molecular Biology, The Pennsylvania State University, University Park, PA 16802, USA.

^4^Department of Molecular Biophysics and Biochemistry, Yale University, New Haven, CT 06520, USA.

*To whom correspondence should be addressed: [r.croce@vu.nl](file:///C:\Users\gisri\Desktop\Apc\r.croce@vu.nl) and [dab14@psu.edu](file:///C:\Users\Riftr\OneDrive\Desktop\dab14@psu.edu)

^†^These authors contributed equally.

^‡^Present address: Department of Biology, University of California San Diego, La Jolla, CA 92093, USA.

**Supplementary Fig. 1** Schematic overview of the vectors involved in the calculation of the CD and OD signals.

**Supplementary Fig. 2** Absorption spectra for allophycocyanin subunits in complexes and isolated subunits.

**Supplementary Fig. 3** Sequence alignment of subunits from FRL-AP, AP, and AP-B.

**Supplementary Fig. 4** Nomenclature of PBP helices.

**Supplementary Fig. 5** Emission spectrum of FRL-AP dissociated by treatment with 3.0 M NaSCN.

.


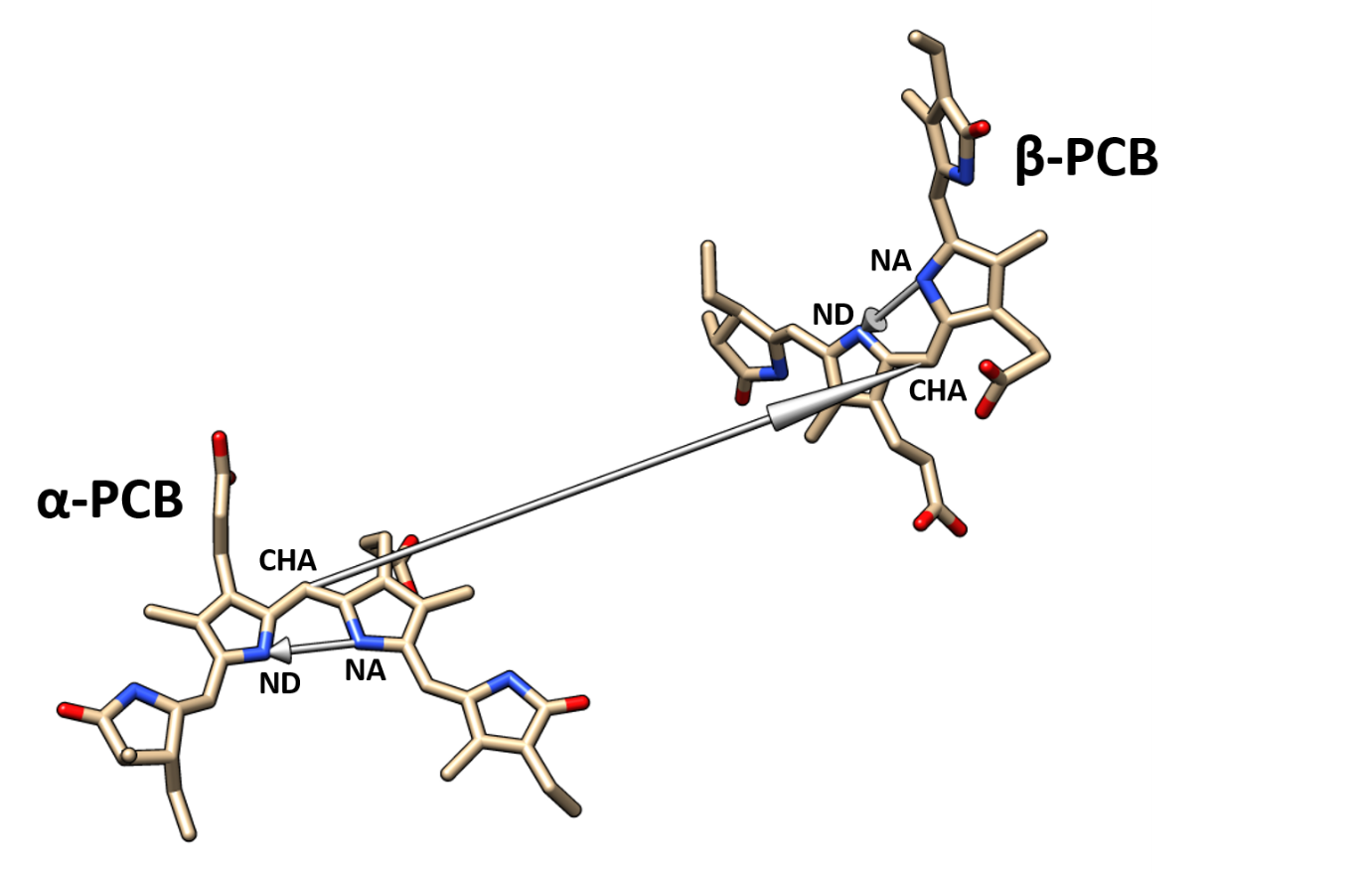


**Supplementary Fig. 1** Schematic overview of the vectors involved in the calculation of the CD and OD signals. The transition dipole moments are directed from the NA to the ND atom for both PCBs. The center-to-center vector is directed between the CHA atom of each PCB.


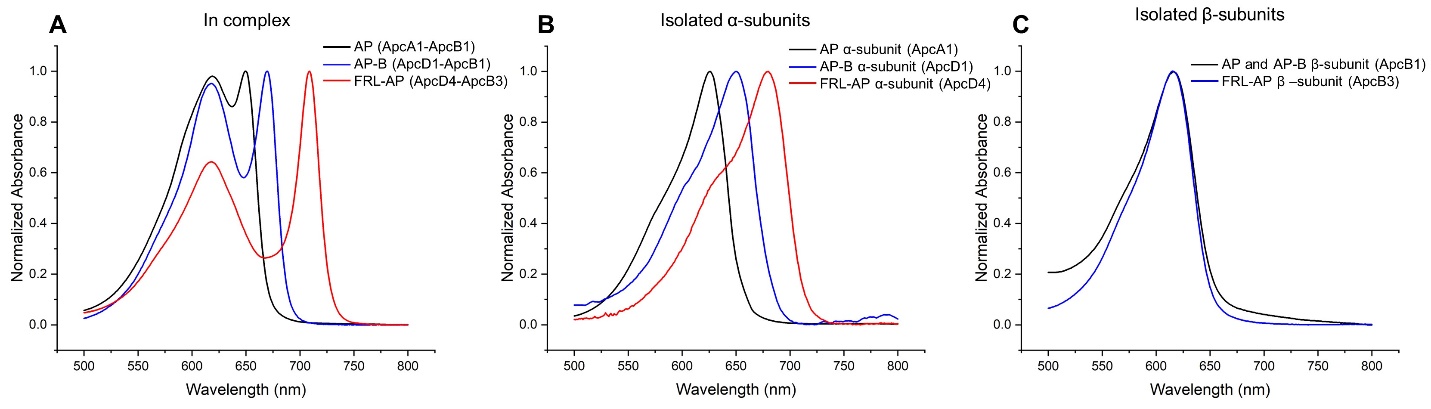


**Supplementary Fig. 2** Absorption spectra for AP complexes and their isolated subunits. **A** Absorbance spectra of oligomeric AP, AP-B, and FRL-AP complexes. Trimeric AP (ApcA1-ApcB1) was produced from the genes of *Leptolyngbya* sp. JSC-1 expressed in *E. coli*. Trimeric AP-B (ApcD1-ApcB1) and helical FRL-AP (ApcD4-ApcB3) were produced from *Synechococcus* sp. A1463 genes. The proteins were produced in and isolated from *E. coli* and *Synechococcus* sp. PCC 7002, respectively. **B** Absorbance spectra of isolated α-subunits. The AP α-subunit (ApcA1) from *Synechocystis* sp. PCC 6803 was expressed and isolated from *E. coli* (Zhang et al. 2009). The AP-B and FRL-AP α-subunits (ApcD1 and ApcD4, respectively) from *Synechococcus* sp. A1463 were produced in and isolated from *E. coli* and *Synechococcus* sp. PCC 7002, respectively. **C** Absorbance spectra of isolated β-subunits. The AP β-subunit (ApcB1) of *Leptolyngbya* sp. JSC-1 was expressed and isolated from *E. coli*. The ApcB3 subunit of *Synechococcus* sp. A1463 was produced in and isolated from *Synechococcus* sp. PCC 7002 (see Soulier and Bryant (2021) for additional details).

A

FRL-AP MSIVAQVIAQSDAADRFLSSAEIAKLEDFFSKGQVRIRAAQKLAENEQKIVQEGSKRFWA 60

AP MSIVTKSIVNADAEARLYSPGELDRIKGFVTSGERRLRIAQVLTESRECIVKQAGDQLFQ 59

AP-B MSVVSQVILQADDQLRYPTSGELKGIQAFLTTGAQRIRIAETLAENEKKIVDQAQKQLFK 60

**:*:: * ::* * : .*: :: *.:.* *:* *: *:*..: **.:. .:::

FRL-AP KCPNTPSNKGNP---QKTAL**C**QRDQGWYIRLVSYCILAGNDKPLEDIGLNGMREMYISLG 117

AP KRPDVVSPGGNAYGEEMTAT**C**LRDMDYYLRLITYGVVAGDVTPIEEIGLVGVREMYNSLG 119

AP-B KHPEYRAPGGNAYGQRQYNQ**C**LRDYGWYLRLVTYGVLAGNKEPIETTGLIGVKEMYNSLN 120

* *: : ** . * ** .:*:**::* ::**: *:* ** *::*** **.

FRL-AP VPLPNLRVAMSCLKEVAAGILSSEEMALAAPYFDRLIRAF- 157

AP TPIPAVAEAVRCMKSVASSLLSGENAAEAASYFDYVVGAMQ 160

AP-B VPVPGMVDAVTVLKDAALGLLSAEDANETAPYFDYIIQFMS 161

.*:* : *: :*..* .:**.*: :* *** :: :

B

FRL-AP MKDTITSLINPADEKGSYLDAAALEQLNRYFQSGNMRVKAAKTISSSASSIISKTVAKSL 60

AP MQDAITAVINSSDVQGKYLDGSAMEKLKAYFQTGKLRVRAATTISANAAEIVKDAVAKSL 60

AP-B MQDAITAVINSADVQGKYLDGAAMDKLKSYFASGELRVRAASVISANAATIVKEAVAKSL 60

*:*:**::** :* :*.***.:*:::*: ** :*::**:**..**:.*: *:..:*****

FRL-AP LYGDITLPGGNMYPTRRYAA**C**LRDLTYFLRYATYAMLAADPSILDERVLQGLKETYITLG 120

AP LYSDITRPGGNMYTTRRYAA**C**IRDLDYYLRYSTYAMLAGDPSILDERVLNGLKETYNSLG 120

AP-B LYSDVTRPGGNMYTTRRYAA**C**IRDLDYYLRYATYAMLAGDASILDERVLNGLKETYNSLG 120

**.*:* ****** *******:*** *:***:******.* ********:****** :**

FRL-AP VPIDRVIQALNAMKEVLTESLDTEASQEMAVYLDHIIAGLS 161

AP VPVGATVQAIQAMKEVTATLVGADAGKEMGVYFDY------ 155

AP-B VPISSTVQAIQAIKEVTASLVGADAGKEMGVYLDYICSGLS 161

**:. .:**::*:*** : :.::*.:**.**:*:

**Supplementary Fig. 3** Sequence alignment of subunits from FRL-AP, AP, and AP-B. **A** Alignment of α-subunits. **B** Alignment of β-subunits. Clustal Omega sequence conservation identifiers are shown below each position. The Cys residue providing the thioester linkage to the phycocyanobilin chromophore for each subunit is in bold font. The sequences for FRL-AP, AP, and AP-B are from species *Synechococcus* sp. A1463, *Phormidium* sp. A09DM, and *Synechocystis* sp. PCC 6803, respectively.


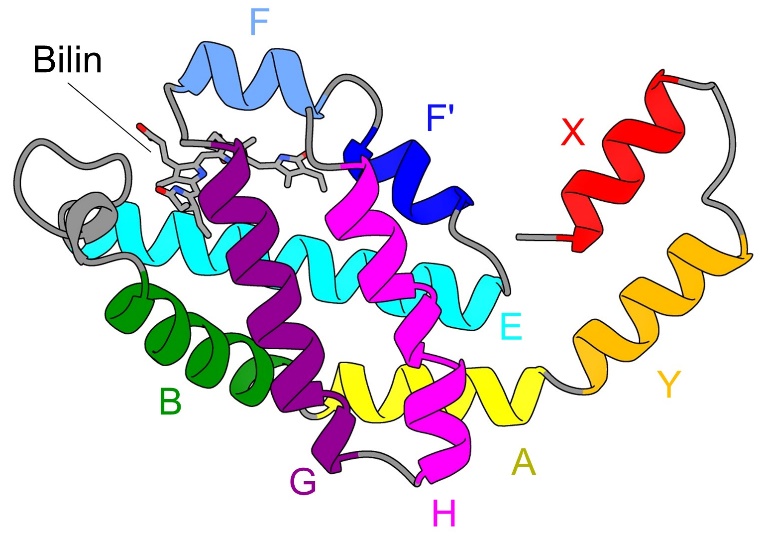


**Supplementary Fig. 4** Nomenclature of PBP helices. The structure of the α-subunit from AP, ApcA1, is shown (Sonani et al. 2015). Helices are labeled as first described in Schirmer et al. (Schirmer et al. 1986). The phycocyanobilin (bilin) chromophore is additionally shown.


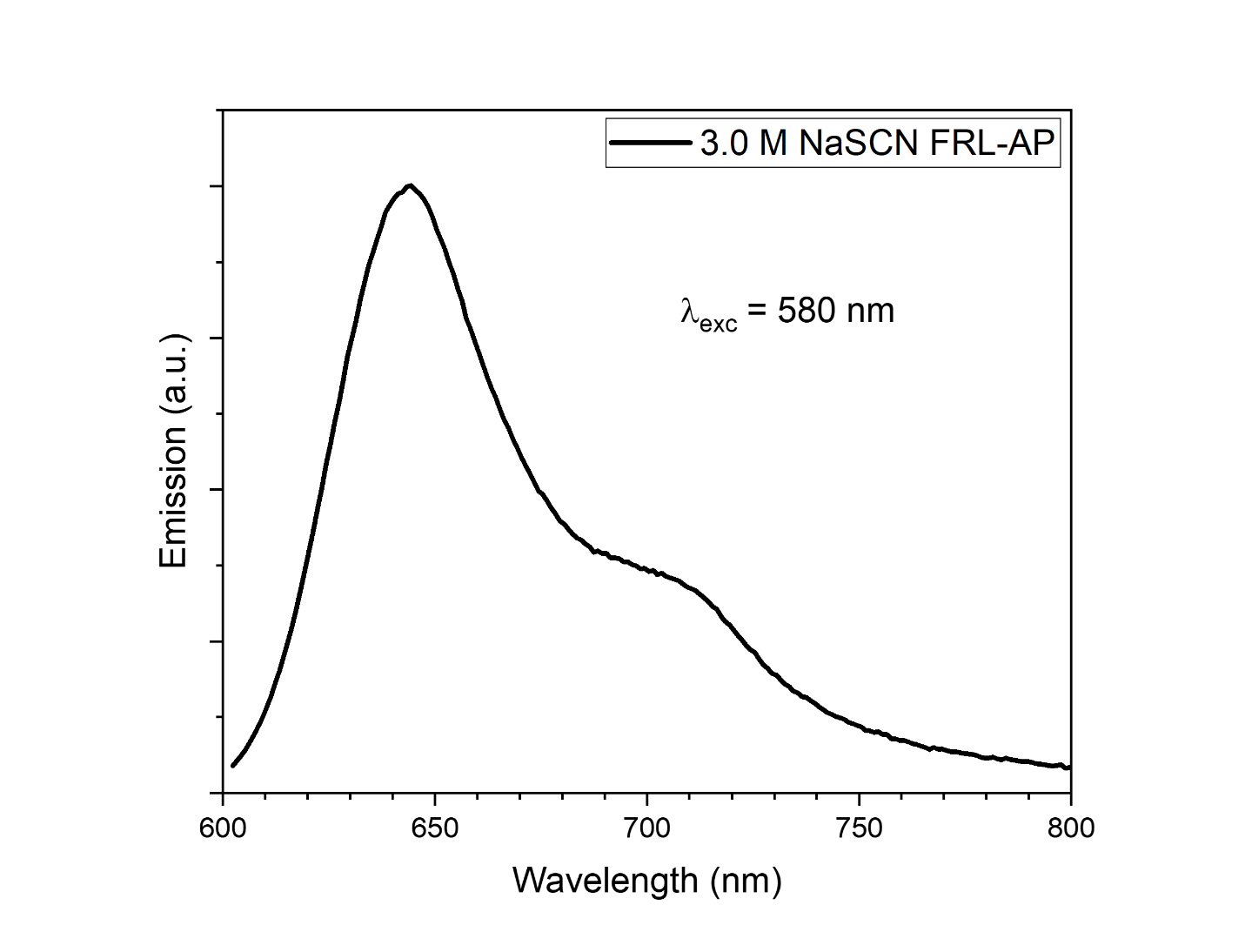


**Supplementary Fig. 5** Emission spectrum of FRL-AP dissociated by treatment with 3.0 M NaSCN. The excitation wavelength was 580 nm.
